# Role of tranexamic acid-soaked gelatin sponge in minimizing rectus sheath hematoma after cesarean section in women treated with warfarin, a simple tool for high-risk cases, a randomized controlled trial

**DOI:** 10.1186/s40001-023-01434-7

**Published:** 2023-10-20

**Authors:** Ayman Hany, Ayman Mansour, Mona Sediek, Mohamed Nabil

**Affiliations:** Obstetrics and Gynecology Department, Faculty of Medicine, Cairo University, Alkasr Alainy St, Cairo, 11562 Egypt

**Keywords:** Tranexamic acid, Gelatin sponge, Rectus sheath hematoma, Anticoagulants

## Abstract

**Background:**

This study aims to illustrate the impact of applying the tranexamic acid impregnated in a gelatin sponge between the anterior rectus sheath and the Rectus Abdominis muscle during Cesarean section (CS) in minimizing rectus sheath hematoma (RHS) in women treated with Warfarin.

**Methods:**

A clinical trial was carried out on 63 pregnant women attended for elective CS, who on antenatal warfarin anticoagulation started from 13 weeks gestation to 36 weeks then shifted to low-molecular-weight heparin (LMWH) or unfractionated heparin (UFH), and with an indication for postnatal warfarin anticoagulation. They were randomly assigned on the day of the scheduled CS into three equal groups (21 women for each). Group 1 had two pieces of gelatin sponges soaked with one ampoule of tranexamic acid. Group 2 had two pieces of gelatin sponges not soaked with tranexamic acid. Group 3 (control group) had no gelatin sponge applied. All patients underwent postoperative assessment done for hemoglobin (Hb), hematocrit (HCT), estimated blood loss (EBL), amount and nature of discharge collected from the sub-rectus drain, complications (RHS, wound infection, thromboembolism), need for re-operation, and need for blood transfusion.

**Results:**

Statistically significant differences were found between Group 1 and Group 2 regarding the postoperative Hb (10.66 ± 1.13 vs. 9.77 ± 0.69, *P* = 0.009), between Group 1 and Group 2 regarding the postoperative HCT (31.87 ± 3.59 vs. 28.54 ± 1.85, *P* = 0.001), between Group 1 and Group 2 regarding EBL (442.19 ± 244.46 vs. 744.38 ± 267.05, *P* = 0.003), between Group 1 and Group 3 regarding EBL (442.19 ± 244.46 vs. 664.29 ± 343.97, *P* = 0.040), and between Group 1 and Group 3 regarding the discharge amount from the sub rectus drain (190.48 ± 100.77 vs. 307.14 ± 127.76, *P* = 0.004).

**Conclusion:**

Tranexamic acid-soaked gelatin sponges are safe and effective in reducing postoperative drainage and EBL.

*Clinical Trial Registration*: At ClinicalTrials.gov in June 2022 (NCT05439694).

## Introduction

Cesarean deliveries are the most common major surgical procedures performed on women globally, and their prevalence rate is gradually increasing in both developed and developing nations [1]. Rectus sheath hematoma (RSH) is a rather uncommon condition that is closely associated with anticoagulant medication. It is still often misdiagnosed despite being well-documented in medical literature for years. Based on the growing usage of anticoagulants, It is assumed that there will be a further increase in usage [2]. RSH occurs due to hemorrhage from the epigastric artery or a tear in the muscle itself. This damage usually occurs as a result of abdominal trauma or an abnormally strong contraction of the Rectus Abdominis muscle. The vessels slide with the changing length of the muscle fibers as they contract to prevent tearing [2].

Regarding the inferior epigastric artery (IEA) tear, it is vulnerable to damage during powerful muscular contractions because of its loose attachment and solid, fixed muscle perforating branches. This explains the higher incidence of RSH occurring in the lower abdomen. On the contrary, bleeding from the superior epigastric artery (SEA) tear usually results in a small hematoma tamponaded by the rectus sheath. Moreover, hematomas induced by IEA tears are less confined because of the anatomical lack of the posterior rectus sheath below the arcuate line of Douglas that may spread outside the midline and posteriorly [2].

Anticoagulant medications are an undeniable risk factor for RSH and may be the most common [3]. In patients receiving anticoagulant medications, the risk of bleeding increases, resulting in increased morbidity and death [4]. Estimates of the total mortality rate from RSH are about 4% and reach up to 25% in individuals receiving anticoagulant therapy [5]. As the prevalence of chemical anticoagulation rises, we can expect the incidence of RHS to follow the same trend. However, modern literature has a scarcity of data to reflect this [6].

Tranexamic acid is a synthetic lysine derivative that suppresses fibrinolysis and clot degradation by attaching to the lysine binding site on plasminogen. It competitively inhibits plasminogen’s enzymatic conversion to plasmin, but at higher concentrations, it acts as a non-competitive inhibitor of plasmin [7].

Topical hemostatic agents can be used as an aide or even an alternative to standard surgical techniques to control bleeding from surgical surfaces. They are principally valuable in instances of diffuse nonanatomic bleeding, bleeding related to delicate structures, and bleeding in patients with abnormalities in hemostasis [8].

Gelatin Sponge is a topical hemostatic intended for application to bleeding surfaces. It is a hydrocolloid synthesized from partial acid hydrolysis of porcine-derived collagen. The gelatin sponge has the capacity to absorb blood or fluid up to 40 times its weight, and it can expand up to 200 percent in its dimensions [8]. The firm, dry sponge form may be customized to any shape; however, it becomes pliable after moistening. The sponge is completely absorbed after four to six weeks [8].

This randomized controlled trial aims to illustrate the impact of applying the tranexamic acid impregnated in a gelatin sponge between the anterior rectus sheath and the Rectus Abdominis muscle during Cesarean section (CS) in minimizing RHS in women treated with Warfarin.

## Methods

According to the CONSORT criteria, a randomized clinical trial was conducted at Kasr Al Ainy Obstetrics and Gynecology Hospital from June 2022 to October 2022 after being approved by the Research Ethics Committee (MS-127-2022). The study was registered at ClinicalTrials.gov in June 2022 (NCT05439694).

**Fig. 1:**
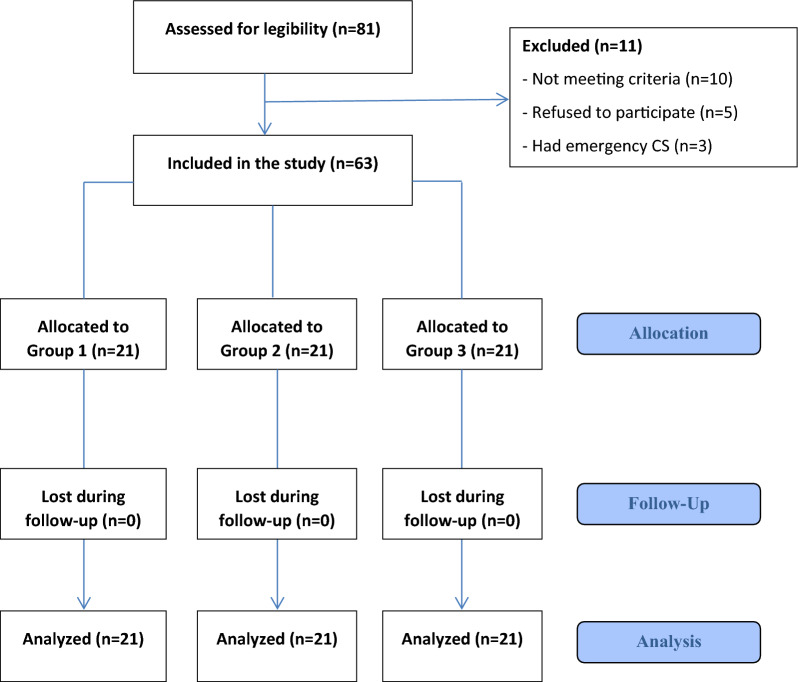
Flow-chart of the study participants

Eighty-one pregnant women who attended the outpatient clinic with an indication for warfarin anticoagulation postnatally were assessed for eligibility. Five patients refused to participate in the study, and ten did not meet our inclusion criteria. Of the remaining 66 patients, three were excluded, being had emergency CS. Therefore, only 63 women were enrolled in our study, as shown in Fig. 1.

The study included pregnant women attended for elective CS, who on antenatal warfarin anticoagulation started from 13 weeks gestation to 36 weeks, then shifted to low-molecular-weight heparin (LMWH) or unfractionated heparin (UFH), and with an indication for postnatal warfarin anticoagulation, e.g., cases of a prosthetic valve, deep venous thrombosis (DVT), dural sinus thrombosis, pulmonary embolism, and atrial fibrillation (AF). The eligibility criteria also included age between 20 and 40 years and body mass index (BMI) between 18.5 and 30.

Women with a risk of obstetric bleeding (i.e., abnormally invasive placenta, placenta previa, placental abruption), renal/hepatic failure, or known allergy to Tranexamic acid were excluded. Women with Hb below 8g/dl were also excluded.

Informed consent was obtained from all participants after being fully informed about the study. Upon admission, precise history taking and thorough clinical examination were conducted for each case, including vital signs, weight, height, and abdominal examination (for assessment of fundal level). Preoperative laboratory investigations, including complete blood count (CBC), as well as abdominal ultrasound, were done for each patient. All enrolled women had elective LSCS by senior obstetricians, using a standardized technique, with visceral and parietal peritoneum re-approximation and inserting a passive intraperitoneal drain.

The participants (*n* = 63) were randomly assigned on the day of the scheduled CS into three equal groups by computer-generated random numbers in opaque envelopes. Group 1 (*n* = 21) included women who had two pieces of gelatin sponges (SURGISPON®; AEGIS LIFESCIENCES, India) (Size, 40 cm^2^) soaked with one ampoule tranexamic acid (Kapron, Amoun Pharmaceuticals SAE, Egypt. 5 ml Amp, 100 mg/ml). Each soaked sponge was applied during CS between the anterior rectus sheath and the Rectus Abdominis muscle (one sponge for each side). Group 2 (*n* = 21) included women who had two pieces of gelatin sponges (SURGISPON^®^; AEGIS LIFESCIENCES, India) (Size, 40 cm^2^) not soaked with tranexamic acid. Each sponge was applied during CS between the anterior rectus sheath and the Rectus Abdominis muscle (one sponge for each side). Group 3 (*n* = 21) included women who had a regular routine CS (neither gelatin sponge nor tranexamic acid was applied).

Fascial closure was done using continuous, slowly absorbable sutures. Meticulous hemostasis was achieved using monopolar cauterization. In all patients, an active drain (Hemovac^®^) was applied in the space between the anterior rectus sheath and the Rectus Abdominis muscle.

All patients were followed up for vital signs and manifestations of allergic reactions in the immediate postoperative period. The intraperitoneal drain was removed after 24 h in all groups. All patients received bridging anticoagulation using LMWH (Enoxaparin) alone for 3 days in therapeutic doses (1 mg/kg twice daily), initiated 12 h after surgery in most cases. Then Warfarin was initiated on postoperative day 3, and LMWH was withdrawn after achieving the target INR.

Finally, the drain was removed, and postoperative assessment was done for hemoglobin (Hb), hematocrit (HCT), estimated blood loss (EBL), amount and nature of discharge collected from the sub-rectus drain, postoperative hospital stay, and incidence of postoperative complications (RHS, wound infection, thromboembolism), need for re-operation, and need for blood transfusion.

The estimated blood loss (EBL) was calculated by the following formula: EBL = EBV x $\frac{\text{Preoperative\,hematocrit}-\,\text{Postoperative\,hematocrit}}{\text{Preoperative\,hematocrit}}$, where EBV is the patient’s estimated blood volume in mL = weight in kg × 85 [9].

Our primary outcomes were the occurrence of RHS and measuring the sub-rectus space drain output. The secondary outcomes were the occurrence of thromboembolic manifestations, the need for postoperative blood transfusion, and the need for surgical management of RHS.

### Sample size calculation

The sample size was calculated using the comparison of drain output during the first eight postoperative hours between women on Warfarin therapy undergoing Cesarean delivery treated with local application of tranexamic acid-impregnated gelatin sponge (G1) and those treated with the application of gelatin sponge alone (G2) compared to non-treated matched women (G3). As reported in a previous publication on spine surgery by Liang et al. [10], the mean ± SD of drain output in G1 was 81.1 ± 61.2 ml, while in G2, it was 166.7 ± 76.8 ml, and in G3, it was 155.7 ± 92.9 ml [10]. As a result, we computed, via G-Power software version 3.1.9.6 (2020) for Microsoft Windows, that 21 women in each group were needed to reject the null hypothesis with 80% power at α = 0.05 level using the One-Way Analysis of Variance test.

### Statistical methods

All statistical analyses were done using Microsoft Excel 2019 (Microsoft Corporation, NY, USA) and IBM SPSS (Statistical Package for the Social Science; IBM Corp, NY, USA) version 25 for Microsoft Windows. Numerical data were statistically described as mean ± standard deviation (± SD), median, and range, while qualitative (categorical) data were described in frequencies (number of cases) and percentages. Student’s t-test was used to compare the numerical variables between the study groups, while Chi-square (χ2) or Exact test was used to compare the categorical data. A probability value (p-value) less than 0.05 was considered statistically significant.

## Results

The study included 63 pregnant women attending cesarean delivery in the high-risk pregnancy unit in Kasr Al Ainy obstetrics and gynecology hospital with an indication for administration of postoperative warfarin anticoagulation. Figure 1 illustrates a summary of the patients’ flow. The demographic data of study participants are summarized in Table 1. There were no statistically significant differences between the groups regarding age, weight, height, BMI, gravidity, parity, number of previous CS, and gestational age at termination.

**Table 1:** Demographic data of study participants

	Group 1 (*n* = 21)	Group 2 (*n* = 21)	Group 3 (*n* = 21)	*P*-value
Age (years)	32.00 ± 4.88 34 (24–39)	30.38 ± 5.84 31 (20–39)	33.05 ± 4.74 34 (26–40)	0.251
Weight (kg)	78.57 ± 4.94 80 (65–85)	78.67 ± 5.29 80 (68–88)	76.14 ± 6.75 78 (65–87)	0.276
Height (cm)	169.62 ± 4.92 170 (160–178)	170.38 ± 4.13 170 (164–177)	168.14 ± 5.20 170 (160–178)	0.31
BMI (kg/m2)	27.37 ± 2.13 27.2 (22.5–30.5)	27.12 ± 2.04 27 (23.5–30.4)	26.93 ± 2.01 26.2 (22.5–31.3)	0.789
Gravidity	3.38 ± 2.16 3 (1–8)	3.33 ± 1.85 4 (0–7)	4.05 ± 2.50 3 (1–11)	0.5
Parity	2.33 ± 1.80 2 (0–7)	2.52 ± 1.47 2 (0–5)	2.57 ± 1.40 3 (1–6)	0.873
Number of Previous CS	1.48 ± 1.29 1 (0–5)	2.24 ± 1.41 2 (0–5)	2.19 ± 1.54 2 (0–6)	0.157
Gestational age (weeks)	37.43 ± 0.68 37 (36–39)	37.43 ± 0.93 37 (35–39)	37.05 ± 0.86 37 (36–39)	0.236

Comparisons between the study groups regarding the preoperative maternal data are shown in Table 2. Regarding the heparin type used before delivery (low-molecular-weight heparin [LMWH] vs. unfractionated heparin [UFH]), there was no statistically significant difference between the groups (*P* = 0.466). Also, there were no significant differences between the groups regarding preoperative hemoglobin (*P* = 0.061) and preoperative hematocrit (*P* = 0.063).

**Table 2:** Preoperative maternal data

	Group 1 (*n* = 21)	Group 2 (*n* = 21)	Group 3 (*n* = 21)	*P*-value
Antenatal heparin				0.466
LMWH	12 (57.14%)	10 (47.62%)	8 (38.10%)	
UFH	9 (42.86%)	11 (52.38%)	13 (61.90%)	
Preoperative Hb	11.35 ± 1.09 11.3 (9.5–13.5)	10.68 ± 0.80 10.7 (9–12.1)	11.29 ± 0.91 11.3 (9.5–13)	0.061
Preoperative HCT	34.10 ± 3.35 33.5 (28.2–41)	32.18 ± 2.25 32.4 (27.9—36.1)	33.85 ± 2.71 33.2 (29.7—39.3)	0.063

Table 3 summarizes the postoperative maternal data. There were significant differences between the three groups regarding the postoperative Hb, postoperative HCT, EBL, and discharge amount from the sub rectus drain. Therefore, we did a second analysis (Tukey HSD posthoc test) to detect the significance between which groups. Statistically significant differences were found between Group 1 and Group 2 regarding the postoperative Hb (10.66 ± 1.13 vs. 9.77 ± 0.69, *P* = 0.009), between Group 1 and Group 2 regarding the postoperative HCT (31.87 ± 3.59 vs. 28.54 ± 1.85, P = 0.001), between Group 1 and Group 2 regarding EBL (*P* = 0.003), between Group 1 and Group 3 regarding EBL (*P* = 0.040), and between Group 1 and Group 3 regarding the discharge amount from the sub rectus drain (*P* = 0.004).

**Table 3:** Postoperative maternal data

	Group 1 (*n* = 21)	Group 2 (*n* = 21)	Group 3 (*n* = 21)	*P*-value
Postoperative Hb	10.66 ± 1.13 10.5 (8.8–12.4)	9.77 ± 0.69 10 (8.4–11.2)	10.38 ± 0.94 10.2 (8.7–11.9)	0.01*
Postoperative HCT	31.87 ± 3.59 32.1 (26.2–37.6)	28.54 ± 1.85 28.9 (24.8–32.6)	30.34 ± 2.76 30.7 (26.1–35.4)	0.001*
EBL (ml)	442.19 ± 244.46 405 (164–1133)	744.38 ± 267.05 736 (174–1206)	664.29 ± 343.97 713 (100–1188)	0.004*
Intraperitoneal drain (ml)	121.43 ± 51.41 100 (50–200)	147.62 ± 67.96 150 (50–300)	166.67 ± 76.38 150 (50–300)	0.092
Sub rectus drain (ml)	190.48 ± 100.77 200 (50–450)	230.95 ± 107.79 200 (100–450)	307.14 ± 127.76 350 (100–500)	0.005*
Sub rectus discharge				
Serous	3 (14.29%)	2 (9.52%)	0 (0.00%)	0.085
Sero-sanguineous	17 (80.95%)	16 (76.19%)	14 (66.67%)	
Bloody	1 (4.76%)	3 (14.29%)	7 (33.33%)	
Postoperative hospital stay (days)	7.62 ± 0.97 8 (6–10)	7.62 ± 0.97 8 (6–10)	7.76 ± 1.04 8 (6–10)	0.867
Postoperative complication				
Rectus sheath hematoma	0 (0.00%)	0 (0.00%)	1 (4.76%)	362
Wound infection	0 (0.00%)	0 (0.00%)	1 (4.76%)	362
Thromboembolism	0 (0.00%)	0 (0.00%)	0 (0.00%)	NA
Need for re-operation	0 (0.00%)	0 (0.00%)	0 (0.00%)	NA
Need for blood transfusion	0 (0.00%)	0 (0.00%)	0 (0.00%)	NA

^*^p-value is significant

The discharge type from the sub rectus drain (serous, serosanguinous, bloody), postoperative hospital stay, postoperative complications (RHS, wound infection, thromboembolism), allergy to tranexamic acid, need for re-operation, and need for blood transfusion were similar in the three groups with no significant difference.

## Discussion

Our study aimed to illustrate the impact of applying the tranexamic acid impregnated in a gelatin sponge between the anterior rectus sheath and the Rectus Abdominis muscle during CS in minimizing RHS in women treated with Warfarin. We found that tranexamic acid-soaked absorbable gelatin sponge reduces the incidence of rectus sheath hematoma formation and decreases the need for blood transfusion among high-risk women undergoing cesarean delivery.

RHS was first reported in the literature by Richardson et al. [11]. Despite being well documented, it is frequently misdiagnosed. RHS represents less than 2% of all patients admitted to the hospital with acute abdominal pain alone; however, it should not be ignored due to its complications that may lead, in some instances, to mortality [2].

The ACOG committee in 2020 stated that there are limited data on the use of topical hemostatic agents in gynecologic and obstetric surgery and, therefore, recommendations are principally created upon findings extrapolated from studies on the employment of these agents in nongynecological and non-obstetric surgeries.

Anticoagulant use has been recognized as the most commonly described risk factor for RSH. Despite that, many trials assessed the efficacy of tranexamic acid in preventing postpartum hemorrhage. To our knowledge, none particularly evaluated its role in patients undergoing CS treated with postnatal Warfarin or its combination (Impregnation) in gelatin sponge to minimize its theoretical systemic adverse effects, especially in high-risk predisposed patients. Therefore, the strength of this randomized control trial is not only that it is the first study to evaluate this simple tool and deal with a serious complication that may occur in those high-risk predisposed cases.

Intravenous use of tranexamic acid following major surgery reduced the requirement for blood transfusion by 32–37% and postoperative hemorrhage by about 34% [12]. The gelatin sponge is a bendable, hydrophilic, absorbable sponge prepared from purified porcine gelatin and is considered a physical matrix for hemostasis [13].

We found out that tranexamic acid-soaked gelatin sponges in Group 1 (combined group) aided in a statistically significant lower EBL when compared to either the gelatin sponge alone group (*p* = 0.003) or the control group (*p* = 0.040). Moreover, Group 1 had a statistically significant lower sub-rectus drain output when compared to Group 3 (*p* = 0.004). Also, Group 1 had a relatively lower output from the sub-rectus space drain than Group 2, yet it was not statistically significant.

In agreement with our results, Liang et al. [10] conducted a randomized controlled trial including 90 patients undergoing spine surgery who were randomized into three groups tranexamic acid-soaked gelatin sponge group (combined group), the gelatin-sponge-only group and a control group [10]. They found that tranexamic acid-soaked gelatin sponge had the potential to significantly reduce postoperative drain output witnessed by the duration of drain retention. In their combined group, it was a statistically significantly shorter duration when compared to the other two groups. The length of hospital stay was also shorter in the combined and gel-foam-only groups compared to the control group. Their study differed from ours in the measurement method of the drain output. They recorded the drain output three times per day in 8-h intervals. In their study, the drain was removed when its output was < 30 ml in 8 h. On the other hand, they had no significant differences in terms of EBL between the three groups [10]. The difference in the type of surgery between our study and theirs could explain this.

In addition, Ausen et al. [14] conducted a randomized controlled trial on 30 women undergoing bilateral breast surgery [14]. Women were divided into two groups, one with topical tranexamic acid versus placebo (saline) in the second group. The drain output was evaluated 24 h following surgery, and the pain was recorded after 3 and 24 h. Postoperative issues were noted, including suture hypersensitivity, seroma, re-bleeding, and wound infection. Agreeing with our results, they concluded that topical application of dilute tranexamic acid reduced bleeding and drainage fluids.

Nonetheless, their study had weaknesses as it was conducted on patients with a surgical procedure not usually accompanied by major bleeding. In addition, they measured drainage fluid volume only, not blood loss. Moreover, our study had a larger sample size and higher bleeding risk patients. However, both studies lacked drain fluid laboratory analysis and serum tranexamic acid concentration measurements.

Shady et al. [15] studied the effect of the topical application of tranexamic acid in reducing blood loss during and after cesarean delivery. They concluded that topical application of tranexamic acid in the placental bed is associated with lower EBL, a lower risk of postpartum hemorrhage, and a lower blood transfusion rate. They had similar findings to ours in terms of reduction in EBL. Nevertheless, they conducted their study on women diagnosed with placenta previa, which is one of the exclusion criteria in our study [15].

Another study conducted by Raga et al. [16] demonstrated results similar to our results regarding effective bleeding control with a topical hemostatic [16]. Fifty women undergoing abdominal myomectomy were enrolled, and the patients were randomized into two groups (*n* = 25). They compared the use of Floseal, a gelatin thrombin matrix solution, versus a control group. They demonstrated a statistically significant reduction in intraoperative blood loss in the Floseal group (*p* < 0.005). Twenty percent of the patients in the control group required blood transfusion compared to none in the Floseal Group (*p* < 0.0001). The average postoperative blood loss assessed by surgical drains at 48 h was significantly reduced in the Floseal group. Moreover, the postoperative hospital stay was significantly lower in the Floseal group (2–3 days) than in the control group (3–7 days). Their study differed from ours in that they included non-pregnant patients undergoing myomectomy and excluded patients on anticoagulation therapy.

The strength of our study is that we combined these concepts using tranexamic acid and an absorbable gelatin sponge placed in the space between Rectus Abdominis and the rectus sheath in pregnant women treated with Warfarin and undergoing CS. On the contrary, the study’s limitations included the relatively small sample size and that the study was restricted to patients undergoing elective CS, excluding obese patients and women with a high risk of massive obstetric bleeding, such as in cases of placenta previa accreta spectrum. Furthermore, we had not undergone laboratory analysis of the drainage fluid, which usually consists of blood and exudates, and we had not measured serum tranexamic acid in patients involved in the study.

## Conclusion

Our study provides an eminent perspective on preventing RHS after CS in Warfarin anticoagulated patients. Tranexamic acid-soaked absorbable gelatin sponge is a safe and effective modality for reducing postoperative drainage and blood loss that may aid in preventing RHS and decrease the need for blood transfusion among high-risk women undergoing cesarean delivery. Further research is needed on a larger scale to emphasize our findings, as well as to include patients with other risk factors for bleeding.

## Data Availability

The data that support the findings of this study are available from Kasr El-Ainy Hospital, but restrictions apply to the availability of these data, which were used under license for the current study, and so are not publicly available. Data are, however, available from the authors upon reasonable request and with permission of Kasr El-Ainy Hospital.
